# Foxp3^+^ T Regulatory Cells as a Potential Target for Immunotherapy against Primary Infection with Echinococcus multilocularis Eggs

**DOI:** 10.1128/IAI.00542-18

**Published:** 2018-09-21

**Authors:** Junhua Wang, Rita Cardoso, Nelson Marreros, Norbert Müller, Britta Lundström-Stadelmann, Myriam Siffert, Dominique A. Vuitton, Franck Boué, Renyong Lin, Hao Wen, Bruno Gottstein

**Affiliations:** a Institute of Parasitology, Department of Infectious Diseases and Pathobiology, Vetsuisse Faculty, University of Bern, Bern, Switzerland; b State Key Lab Incubation Base of Xinjiang Major Diseases Research (2010DS890294) and Xinjiang Key Laboratory of Echinococcosis, First Affiliated Hospital of Xinjiang Medical University, Urumqi, Xinjiang, China; c Central Animal Facilities, Department of Infectious Diseases and Pathobiology, Vetsuisse Faculty, University of Bern, Bern, Switzerland; d WHO-Collaborating Centre on Prevention and Treatment of Human Echinococcosis and French National Reference Centre on Alveolar Echinococcosis, University of Franche-Comté and University Hospital, Besançon, France; e ANSES, Laboratoire de la rage et de la faune sauvage de Nancy, Malzéville, France; Washington State University

**Keywords:** alveolar echinococcosis, regulatory T cells, Th1 immunity, oral infection, Treg deficiency

## Abstract

Alveolar echinococcosis (AE) is a lethal disease caused by infection with the metacestode stage of the helminth Echinococcus multilocularis, which develops into a tumorlike mass in susceptible intermediate hosts. The growth potential of this parasite stage is directly linked to the nature of the surrounding periparasitic immune-mediated processes. In a first step (experiment 1), mice were orally infected with E. multilocularis eggs, to be used for assessing the hepatic expression profiles of 15 selected cytokine and chemokine genes related to acquired immunity from 21 to 120 days postinfection. The early stage of infection in immunocompetent animals was marked by a mixed Th1/Th2 immune response, as characterized by the concomitant presence of gamma interferon (IFN-γ) and interleukin-4 (IL-4) and their related chemokines. At the late stage of AE, the profile extended to a combined tolerogenic mode including Foxp3, IL-10, and transforming growth factor beta (TGF-β) as key components. In a second step (experiment 2), the effect of T regulatory cell (Treg) deficiency on metacestode growth was assessed in E. multilocularis-infected DEREG (depletion of regulatory T cells) mice upon induction of Treg deficiency with diphtheria toxin (DT). The parasite lesions were significantly smaller in the livers of treated mice than in corresponding control groups. Foxp3^+^ Tregs appear to be one of the key players in immune-regulatory processes favoring metacestode survival by affecting antigen presentation and suppressing Th1-type immune responses. For these reasons, we suggest that affecting Foxp3^+^ Tregs could offer an attractive target in the development of an immunotherapy against AE.

## Abstract (precis)

Alveolar echinococcosis (AE) is a lethal disease caused by infection with the metacestode stage of the helminth Echinococcus multilocularis, which develops into a tumorlike mass in susceptible intermediate hosts. The growth potential of this parasite stage is directly linked to the nature of the surrounding periparasitic immune-mediated processes.

## INTRODUCTION

Alveolar echinococcosis (AE) is a severe zoonotic helminth disease, which is fatal to patients if not appropriately diagnosed and treated (1). The adult tapeworms of Echinococcus multilocularis persist in the intestine of their final hosts (mainly canids, such as foxes, dogs, raccoon dogs, and coyotes) and produce infectious eggs that are released into the environment with the feces. Once these eggs are ingested by intermediate hosts (i.e., rodents, and accidentally humans), they hatch, and released oncospheres penetrate through the small intestinal mucosa. From there, they are transported via portal blood flow to the liver. Here the larvae (metacestodes) establish within the liver parenchyma, with a subsequent continuous growth and maturation process. The larval mass gradually proliferates and infiltrates the liver parenchyma, thus destroying the surrounding host tissue, and resembles a slow-growing malignant neoplasm in both its macroscopic appearance and behavior (2). The disease AE is thus caused by a chronically progressing hepatic damage due to the continuous proliferation of the metacestode (larval stage) of E. multilocularis in a tumorlike way (3). The metacestode tissue is also able to metastasize, thus also progressively invading other sites such as lungs and brain, among others (4). The kind and type of the immune response elicited by E. multilocularis infection have an impact on the development of clinical signs and outcome of disease (2, 3), ranging from resistance (self-cure) to high susceptibility characterized by evolving disease and subsequent fatality (4). Human patients with a Th2-orientated immunity are more likely to develop chronic AE, while those with Th1 cell activation more commonly show reduced or even aborted parasites (visible as died-out lesions) and thus protection (3, 4). In human AE, CD4^+^ CD25^+^ T regulatory cells (Tregs), which play a major role in the regulation of immune response, appeared to be upregulated during the time course of the disease. This upregulation appears to be associated with the blunting of immune responses to specific antigens and/or to the suppression of the secretion of proinflammatory cytokines, especially through high interleukin-10 (IL-10) and transforming growth factor beta 1 (TGF-β1) production (4).

The most common experimental infection model of AE so far is provided by the secondary murine infection model. In this model, the infection is induced through the intraperitoneal (i.p.) or intrahepatic injection of E. multilocularis metacestode tissue suspension. The immune reaction is characterized mainly by a Th1-oriented response during the early stage of infection (approximately until 1 month postinfection [p.i.]), which gradually shifts toward a more dominant Th2-oriented response within the 2 to 4 subsequent months but still remains a mixture of Th1/Th2 within the chronic phase of infection. During this chronic phase, proinflammatory cytokines are expressed in the periparasitic granuloma. The infected mice show a partial/relative protective immunity mainly by means of periparasitic liver fibrosis and central parasite tissue necrosis that lead to a restricted parasite growth (5, 6). Also, CD4^+^ CD25^+^ Tregs appeared to act as a central immune cell population in triggering periparasitic anergy (7). In a similar context, E. multilocularis-infected DEREG (depletion of regulatory T cells) mice exhibited a significantly lower parasite mass/load when they were treated with diphteria toxin (DT) to induce Treg deficiency than non-DT-treated controls (8). However, the natural mode of infection, or primary infection, of intermediate hosts with E. multilocularis results from peroral uptake of infectious eggs, and unlike the above-described secondary infection model, this peroral infection includes also the first phase of intrahepatic (postoncospheral) parasite development, which might be crucial in determining the early immune response during AE. Such a model is seldom applied in experimental infection due to the high risk for the staff and thus the necessity of working in a biosafety level 3 facility. Some studies used a primary infection model, but these were restricted to the description of conventional humoral immune reactions (9, 10) or of morphological growth behavior in different mouse strains (11, 12). No study has reported on the host immune response in primary murine AE, especially not addressing the immunomodulatory events induced by the parasite and its subsequent effect on the host immune response.

The objectives of this study were to apply the mouse model of primary E. multilocularis infection such as (i) to elucidate immunological key parameters occurring under primary infection and (ii) to explore whether Foxp3^+^ Tregs could be putatively tackled as an immunotherapeutic target against primary E. multilocularis infection.

## RESULTS

To get a more complete picture on the influence of the parasite-induced host immune response following primary infection, in experiment 1 the expression levels of 15 key cytokines and chemokines were measured in the spleen applying reverse transcription-quantitative PCR (qRT-PCR) at 21, 45, 60, and 120 days p.i.

### Inflammatory and proinflammatory cytokines.

**FIG 1:**
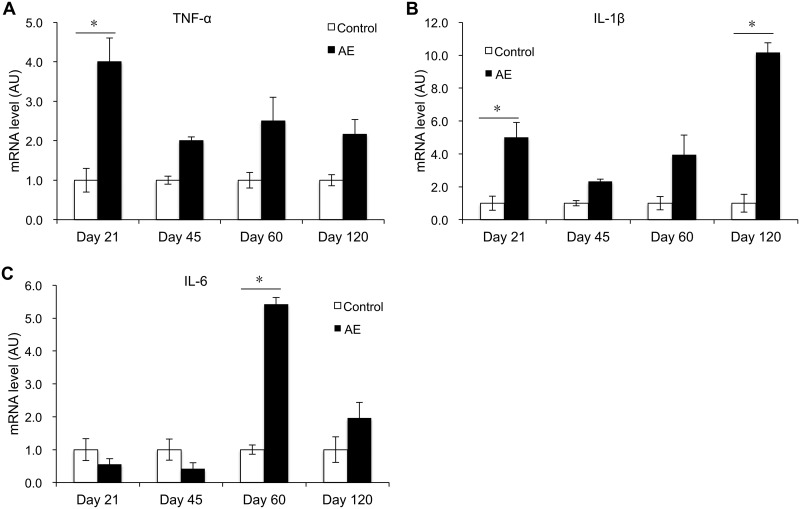
Course of proinflammatory cytokine gene expression in the spleen of mice following peroral E. multilocularis infection (measured by qRT-PCR). (A) TNF-α; (B) IL-1β; (C) IL-6. Data represent means ± SD from three independent experiments with a total of 18 mice in each group (6 mice per group in each independent experiment). Comparison between groups was performed using a two-way ANOVA with Bonferroni's multiple comparison posttest for statistical analysis. *, *P* < 0.05. Control, noninfected mice; AE, E. multilocularis-infected mice; AU, arbitrary units.

Mice primarily infected with E. multilocularis eggs showed significant differences during the course of proinflammatory cytokine development in the spleen during 21 to 120 days (sampling days 21, 45, 60, 120) of expressional assessment by real-time quantitative PCR (RT-qPCR), compared with noninfected control mice: tumor necrosis factor alpha (TNF-α) mRNA expression was increased at day 21 p.i. (*P* < 0.05). Subsequently, it rapidly decreased and maintained a low expression level during day 45 and day 120 p.i. (Fig. 1A). IL-1β mRNA expression was significantly higher than in control mice at day 21 p.i. (*P* < 0.05), dropped at day 45 p.i., reincreased, and reached its maximum at day 120 p.i. (*P* < 0.05). Infected mice displayed a significant increase in IL-6 mRNA expression at day 60 p.i. (*P* < 0.05) (Fig. 1C).

### Th1/Th17 cytokines and related chemokines. (i) Th1-related cytokines and chemokines.

**FIG 2:**
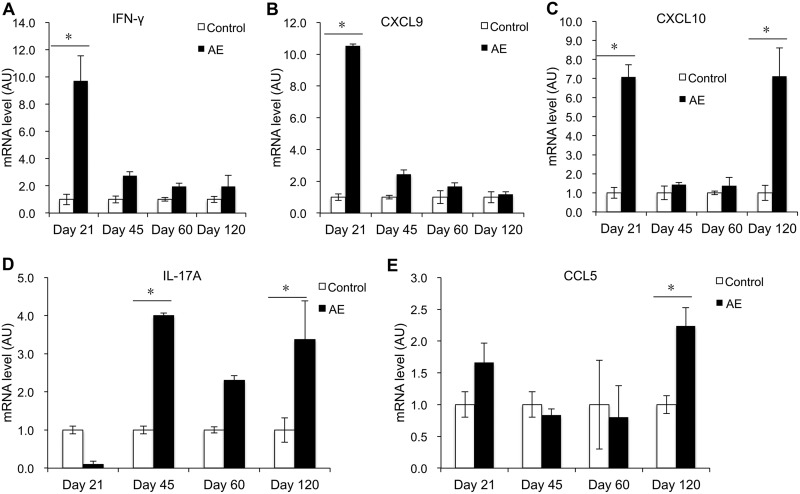
Course of Th1/Th17-related cytokine and chemokine gene expression in the spleen of mice following peroral E. multilocularis infection (measured by qRT-PCR). (A) IFN-γ; (B) CXCL-9; (C) CXCL-10; (D) IL-17A; (E) CCL-5. Data represent means ± SD from three independent experiments with a total of 18 mice in each group (6 mice per group in each independent experiment). Comparison between groups was performed using a two-way ANOVA with Bonferroni's multiple comparison posttest for statistical analysis. *, *P* < 0.005. Control, noninfected mice; AE, E. multilocularis-infected mice; AU, arbitrary units.

In the spleen of infected mice, gamma interferon (IFN-γ) mRNA expression was significantly increased at day 21 p.i. (*P* < 0.05). It subsequently decreased to normal levels during days 45 to 120 p.i. (Fig. 2A). In parallel with IFN-γ, the level of CXCL9 mRNA expression in infected mice was markedly higher at day 21 p.i. (*P* < 0.05) (Fig. 2B). The expression level of CXCL10 mRNA was also markedly increased at day 21 p.i. (*P* < 0.05), but it was increased again at day 120 p.i. despite a subsequent drop at days 45 and 60 p.i. (Fig. 2C).

### (ii) Th17-related cytokines and chemokines.

In the spleen of infected mice, IL-17A mRNA expression increased and then remained at a high expression level at days 45 and 120 p.i. (*P* < 0.05), with a relative decrease at day 60 p.i. (Fig. 2D). However, CCL5 mRNA expression was significantly increased only at day 120 p.i. compared to control mice (*P* < 0.05) (Fig. 2E).

### Th2 cytokines and related chemokines.

**FIG 3:**
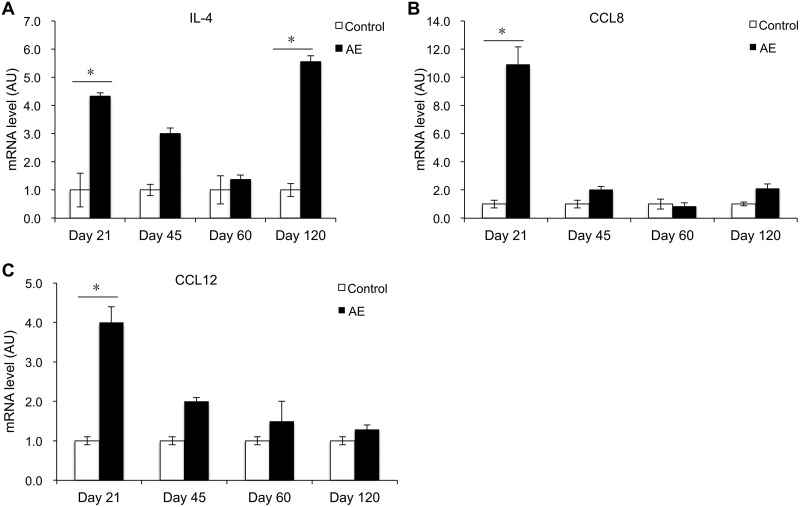
Course of Th2-related cytokine and chemokine gene expression in the spleen of mice following peroral E. multilocularis infection (measured by qRT-PCR). (A) IL-4; (B) CCL-8; (C) CCL-12. Data represent means ± SD from three independent experiments with a total of 18 mice in each group (6 mice per group in each independent experiment). Comparison between groups was performed using a two-way ANOVA with Bonferroni's multiple comparison posttest for statistical analysis. *, *P* < 0.05. Control, noninfected mice; AE, E. multilocularis-infected mice; AU, arbitrary units.

In the spleen of infected mice, IL-4 mRNA expression was increased at day 21 p.i. (*P* < 0.05), compared to controls. IL-4 subsequently decreased and then remained at a low expression level around the middle stage of E. multilocularis infection. It reincreased markedly at day 120 p.i. (*P* < 0.05) (Fig. 3A). However, CCL8 and CCL12 mRNA expression was significantly increased only at day 21 p.i. (*P* < 0.05) (Fig. 3B and C).

### Treg-related nuclear transcription factor and cytokines.

**FIG 4:**
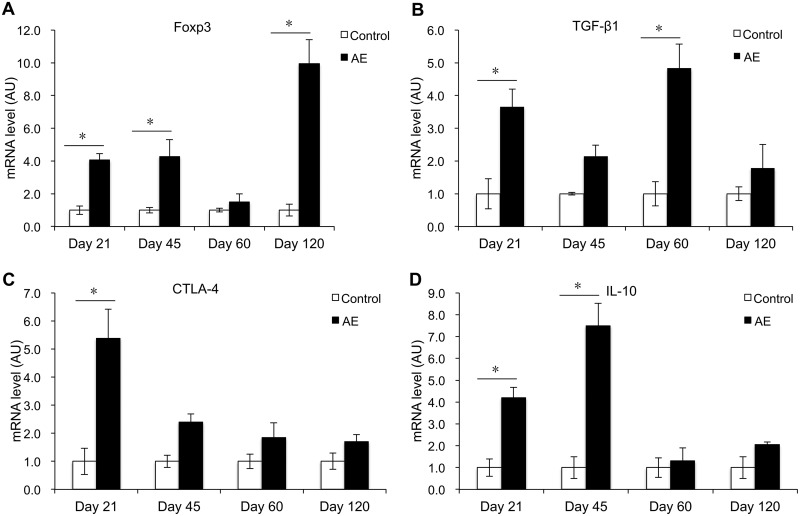
Course of Treg nuclear transcription factor and cytokine gene expression in the spleen of mice following peroral E. multilocularis infection (measured by qRT-PCR). (A) Foxp3; (B) TGF-β1; (C) CTLA-4; (D) IL-10. Data represent means ± SD from three independent experiments with a total of 18 mice in each group (6 mice per group in each independent experiment). Comparison between groups was performed using a one-way ANOVA with Bonferroni's multiple comparison posttest for statistical analysis. *, *P* < 0.05. Control, noninfected mice; AE, E. multilocularis-infected mice; AU, arbitrary units.

In the spleen of infected mice, Foxp3 mRNA expression was increased at days 21 and 45 p.i., compared to controls. After a drop at day 60 p.i., Foxp3 expression was markedly reincreased at day 120 p.i. (*P* < 0.05) (Fig. 4A). TGF-β1 mRNA expression was increased at days 21 and 45 p.i. (*P* < 0.05) (Fig. 4B), while CTLA-4 mRNA expression was significantly increased at day 21 p.i. (*P* < 0.05) (Fig. 4C). IL-10 mRNA expression was significantly increased at 21 and 45 days p.i. (*P* < 0.05).

### Effect of E. multilocularis egg infection on serum cytokine levels.

**FIG 5:**
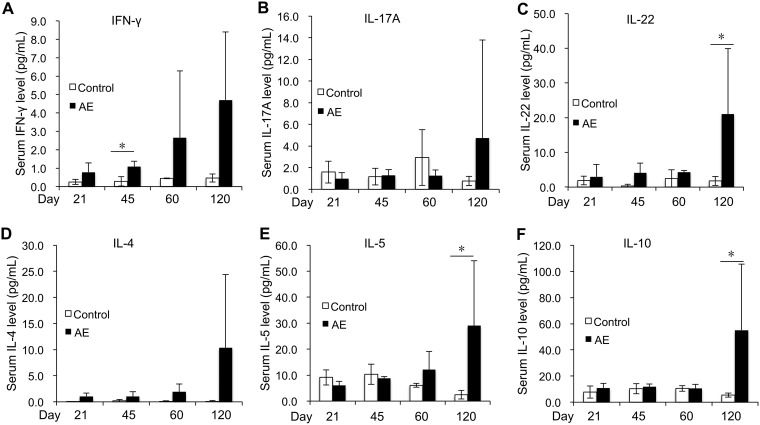
Course of Th-related serum cytokine expression levels following peroral E. multilocularis infection (measured by mesoscale). (A) IFN-γ; (B) IL-17A; (C) IL-22; (D) IL-4; (E) IL-5; (F) IL-10. Data represent means ± SD for 6 mice in each group. Comparison between groups was performed using a two-way ANOVA with Bonferroni's multiple comparison posttest for statistical analysis. *, *P* < 0.05. Control, noninfected mice; AE, E. multilocularis-infected mice.

To determine whether E. multilocularis egg infection impacts on the immune profile of systemic immunity, we measured the concentration of Th-related cytokines in the serum. Thus, the IFN-γ level in serum was increased at day 45 p.i. in infected mice (*P* < 0.05) and then maintained a higher level than in noninfected animals (Fig. 5A). The IL-17A level in serum showed no statistically significant difference between AE mice and controls; however, another Th17-related cytokine, IL-22, was observed to be much higher on day 120 p.i. in AE mice (*P* < 0.05) than in noninfected controls (Fig. 5B and C). With regard to Th2-related cytokines, IL-4 levels in serum showed no statistically significant differences between AE mice and controls, whereas IL-5 yielded higher levels at day 120 p.i. in AE mice (*P* < 0.05) than in noninfected controls (Fig. 5D and E). The IL-10 level in serum was increased at day 120 p.i. in AE mice (*P* < 0.05) compared to noninfected controls (Fig. 5F).

### Foxp3^+^ Tregs as a potential immunotherapeutic target against primary AE.

**FIG 6:**
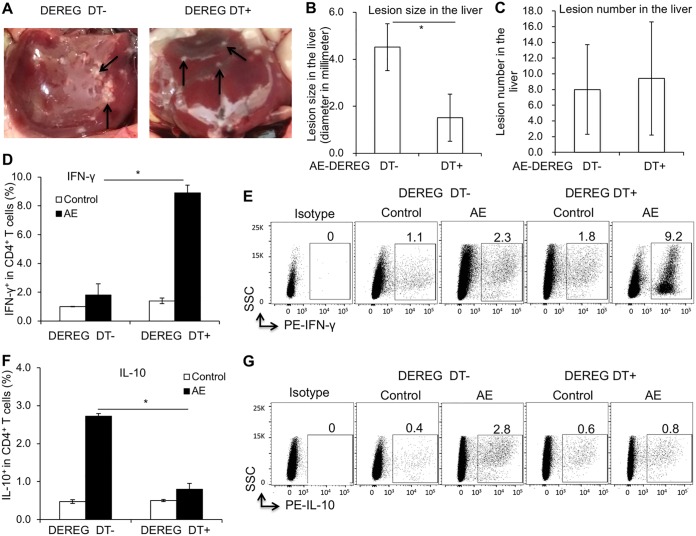
Foxp3^+^ Treg-inducible knockdown as a potential immunotherapeutic target against primary AE- and Th-related immune response (assessed by FACS). (A) Representative picture of individual parasite lesions (indicated by arrows) in the liver of AE-DEREG DT^−^ and AE-DEREG DT^+^ mice at 3 months postinfection. (B) Lesion size in the liver of AE-DEREG DT^−^ and AE-DEREG DT^+^ mice 3 months postinfection. (C) Number of lesions in the liver from AE-DEREG DT^−^ and AE-DEREG DT^+^ mice 3 months postinfection. (D) Frequency of IFN-γ^+^ T cells within CD4^+^ T cells in spleen cells from AE-DEREG DT^−^ and AE-DEREG DT^+^ mice at 3 months postinfection. (E) Representative scatter plots of IFN-γ^+^ T cells within CD4^+^ T cells in spleen cells from AE-DEREG DT^−^ and AE-DEREG DT^+^ mice 3 months postinfection. (F) Frequency of IL-10^+^ T cells within CD4^+^ T cells in spleen cells from AE-DEREG DT^−^ and AE-DEREG DT^+^ mice. (G) Representative scatter plots of IL-10^+^ T cells within CD4^+^ T cells in spleen cells from AE-DEREG DT^−^ and AE-DEREG DT^+^ mice 3 months postinfection. Data represent means ± SD for a total of 6 mice in the noninfected control group and 5 mice in the AE group (AE-DEREG DT^−^ and AE-DEREG DT^+^) after excluding the mice with subcutaneous lesions or no lesion in the liver. Comparison between groups was performed using a two-way ANOVA with Bonferroni's multiple comparison posttest for statistical analysis. *, *P* < 0.025. DEREG DT^−^, *foxp3*-inducible knockdown mice (DEREG mice) without DT application; DEREG DT^+^, DEREG mice with DT application; AE-DEREG DT^−^, E. multilocularis-infected DEREG without DT application; AE-DEREG DT^+^, E. multilocularis-infected DEREG mice with DT application. Control, noninfected mice. DT application started 1 month postinfection and was maintained for 1 month, and the mice were sacrificed at 3 months postinfection.

Experiment 2 focused on the potential role of Foxp3^+^ Tregs in the immunological fight against primary AE. Infected mice with active or depleted Foxp3 expression were assessed for their Th1/Th2-related and Treg/Th17-related cytokines, antigen-presenting cell (APC) activation, and the generation as well as the function of Tregs. Abrogation of Treg function was obtained in infected DEREG mice by DT treatment (intraperitoneal injection, 110 ng/mouse), initiated 3 weeks p.i. and subsequently maintained for 4 weeks (with 3 applications/week). Two mice (one from the treatment group and another from the nontreated group) were excluded for statistical analysis because of subcutaneous lesion. Foxp3^+^ Treg-depleted mice had parasite lesions in the liver that were smaller on average than those of nondepleted controls (Fig. 6A and B). However, the numbers of hepatic lesions were similar in the two groups (Fig. 6C). This can be explained by the fact that depletion took place after the primary establishment of the oncospheres within the liver had occurred.

Th cells from Foxp3^+^ Treg-depleted mice were oriented toward a much stronger Th1 immune response than those from control mice. This appeared as a significantly higher IFN-γ (Fig. 6D and E) and a lower IL-10 (Fig. 6F and G) production. However, the difference respective to Th2 and Th17 immune response orientation was not significant (these data are supported by Fig. S1 in the supplemental material).

**FIG 7:**
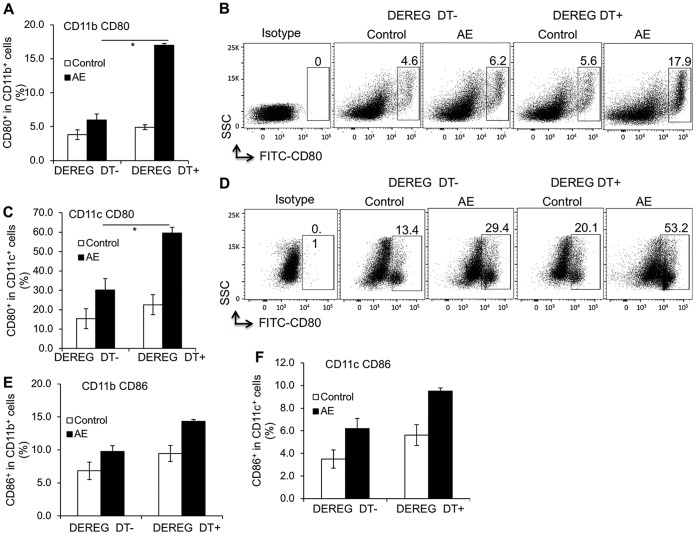
Foxp3^+^ Treg-inducible knockdown against primary AE and its related APC activation (assessed by FACS). (A) CD80 frequency within CD11b^+^ APCs in spleen cells from AE-DEREG DT^−^ and AE-DEREG DT^+^ mice at 3 months postinfection. (B) Representative scatter plots of CD80^+^ cells within CD11b^+^ APCs in spleen cells from AE-DEREG DT^−^ and AE-DEREG DT^+^ mice at 3 months postinfection. (C) CD80 frequency within CD11c^+^ APCs in spleen cells from AE-DEREG DT^−^ and AE-DEREG DT^+^ mice at 3 months postinfection. (D) Representative scatter plots of CD80^+^ cells within CD11c^+^ APCs in spleen cells from AE-DEREG DT^−^ and AE-DEREG DT^+^ mice at 3 months postinfection. (E) CD86 frequency within CD11b^+^ APCs in spleen cells from AE-DEREG DT^−^ and AE-DEREG DT^+^ mice at 3 months postinfection. (F) CD86 frequency within CD11c^+^ APCs in spleen cells from AE-DEREG DT^−^ and AE-DEREG DT^+^ mice at 3 months postinfection. Data represent means ± SD for a total of 6 mice in noninfected control groups and 5 mice in the AE groups (AE-DEREG DT^−^ and AE-DEREG DT^+^) after excluding mice developing subcutaneous lesions or presenting no lesion at all. Comparison between groups was performed using a two-way ANOVA with Bonferroni's multiple-comparison posttest for statistical analysis. *, *P* < 0.05. DEREG DT^−^, *foxp3*-inducible knockdown mice (DEREG mice) without DT application; DEREG DT^+^, DEREG mice with DT application; AE-DEREG DT^−^, E. multilocularis-infected DEREG without DT application; AE-DEREG DT^+^, E. multilocularis-infected DEREG mice with DT application. Control, noninfected mice. DT application started 1 month postinfection and was maintained for 1 month, and mice were sacrificed at 3 months postinfection.

Both subsets of APCs, CD11b^+^ and CD11c^+^ cells, displayed a higher frequency of CD80 in depleted mice than in controls (Fig. 7A to D). However, no difference in CD86 frequency was present (Fig. 7E and F).

## DISCUSSION

**FIG 8:**
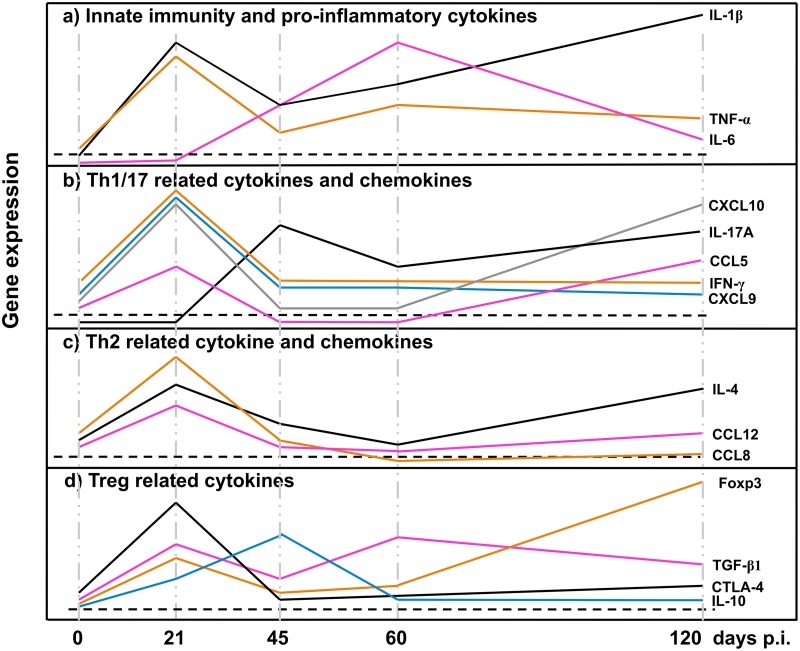
Course of the gene expression of innate immunity and proinflammatory cytokines (a), Th1/Th17-related cytokines and chemokines (b), Th2-related cytokines and chemokines (c), and Treg-related cytokines (d). p.i., postinfection.

The main immunological characteristic upon infection with E. multilocularis leading to AE is a progressively increasing tolerance status of the host, marked by a downregulation of periparasitic immune effector mechanisms. This anergic property develops and increases over time and reaches its maximum at the late stage of disease, both in naturally infected humans (13) and in mice experimentally infected via i.p. or intrahepatic metacestode inoculation (14). However, data on the postinfection cellular immune responses following peroral infection of mice with E. multilocularis eggs, which mimics the natural infection route for human AE patients, have scarcely been published (9). For the first time, we could now show that in mice subjected to primary infection with E. multilocularis eggs (Fig. 8), (i) there was a parallel expression of Foxp3 and CTLA-4 plus IL-10 at a very early stage of infection, (ii) IL-17A was involved at the early/middle stage of the immune response and remained markedly expressed all along the course of infection, (iii) a parallel additional pattern of cytokines and related chemokines indicated their continuous role in maintaining the homing of immune cells at different infection stages, and (iv) most importantly, Foxp3^+^ Tregs were found to be an important immunological factor promoting E. multilocularis proliferative activity in mice. We showed that downregulation of Foxp3^+^ Tregs might be a strategy to reinforce the immunological periparasitic control of intrahepatic E. multilocularis infection.

The messenger RNAs of proinflammatory cytokines TNF-α, IL-1β, and IL-6, which initiate the fibrotic process, have been demonstrated in macrophages located at the periphery of granulomas (15). In the present study, we showed that mRNA levels of TNF-α, IL-1β, and IL-6 in the spleen were developing alternately during the different stages of primary E. multilocularis infection. The initial increase of TNF-α and IL-1β expression with a subsequent dramatic decrease after day 45 p.i. is in accordance with previous reports (5). TNF-α appeared to have a protective role against E. multilocularis (16, 17). Patients with active AE, i.e., a metacestode proliferating in the periphery and occasionally showing central necrosis, also displayed higher expression of TNF-α mRNA in surrounding cells. The activation of pathogen recognition receptors of immune cells, such as monocytes and macrophages, stimulates a range of signaling pathways, including NF-κB, which may enhance the transcription of inflammatory cytokines genes such as those encoding IL-6, TNF-α, and IL-1β. TNF-α and IL-1β also activate transcription factors to produce IL-6 (18). That could explain the early expression of TNF-α and IL-1β in our present study. IL-6 then appeared, presumably to sustain the inflammatory response at the middle infection stage, and possibly also to transmit/represent a warning signal in the event of tissue damage. Secretion of the proinflammatory cytokines IL-1β and IL-18 by peripheral blood mononuclear cells (PBMCs) of AE patients appeared to be reduced in response to E. multilocularis metacestodes, compared to controls (19). In the present study, however, IL-1β was highly expressed at the early stage, subsequently decreased at the middle stage, but reincreased at the late stage. Such selective dynamics of proinflammatory cytokine release may maintain the periparasitic immune infiltrate from the very early stage of infection to the chronic stage.

Several concordant previous observations had shown that PBMCs of AE patients as well as spleen or lymph node cells of experimentally infected mice exhibited a markedly and steadily increasing Th2 orientation of the immune response (20). Our previous studies also documented a biphasic curve of IL-4 mRNA expression, with a very early peak at 2 to 8 days after intrahepatic infection (5), which was also found in the present study. The early expression of IL-4 mRNA might be crucial to prime naive CD4^+^ T cells into differentiated Th2-type cells (21) and to prevent the development of an immune-mediated antiparasitic resistance (22, 23). In the present study, spleen cells displayed an increase of IL-10 mRNA at the early stage of infection, but with a delayed increase of Foxp3 mRNA at the middle stage and a decrease of IFN-γ mRNA at the late infection stage. Furthermore, there was an increase of IL-10 expression in the serum at the late infection stage. This delayed increase of Foxp3/decrease of IFN-γ and increase of serum IL-10 level at the late infection stage match previous observations at the late stage of infection of human AE (24–26) and are in agreement with the data usually reported from lymphocyte studies in secondarily infected mice (27). This combined cytokine profile is highly indicative of a strategy from the parasite to evade host immune response effectors (5, 13, 20).

There is growing evidence that parasite-induced immunomodulation is a hallmark of the pathophysiology of AE (28). Tregs play a dominant role in the suppression of autoimmune pathologies, as rescue of Treg number or function in model systems can both prevent and reverse disease (29). In the mouse, a lack of CD4^+^ Foxp3^+^ Tregs results in increased autoimmunity, and adoptive transfer of Tregs prevents and reverses autoimmunity (30). Overexpression of regulatory cytokine genes in Tregs, such as IL-10 and TGF-β, characterizes immune tolerance in a number of parasitic disease models (31). In AE, earlier studies have shown that CD4^+^ CD25^+^ Tregs were upregulated in peritoneal exudate cells (PECs) from wild-type (WT) E. multilocularis-infected mice (32). The same Treg lines seem also to be upregulated in human AE patients (33). Tregs appear to be one of the key immune subsets to mediate the progressing anergic immune status in chronic infection (5, 34, 35). The smaller AE lesion sizes in the liver of Foxp3^+^ Treg-depleted mice from our study corroborate the importance of Tregs in AE, a phenomenon that was associated with a higher Th1 immune response, a lower IL-10 production, no difference of Th2/Th17 immunity, and upregulation of APC activation.

In our primary infection model, the late infection was characterized by a strong Foxp3 expression and weak expression of most mediators, suggesting that their production is suppressed by Tregs. Understanding how Foxp3^+^ Tregs regulate the immune process in AE could help find new immunotherapeutic targets. Future studies should further explore immunomodulatory parameters such as Foxp3^+^ Treg-blocking agents (i.e., PD1/PD-L1 blockade). The final aim should be to convert the immunological anergy during chronic disease into a more proactive, Th1-oriented immunity, with potential reduction or even inactivation of the metacestode tissue, and thus a healing option for the host. Such an approach could even be coupled to a parasitostatic benzimidazole medication in order to obtain therapeutic synergy.

## MATERIALS AND METHODS

### Ethics statement.

The animal study was performed in strict accordance with the recommendations of the Swiss Guidelines for the Care and Use of Laboratory Animals. The protocol was approved by the governmental Commission for Animal Experimentation of the Canton of Bern (approvals no. BE103/11 and BE112/14).

### Experimental design, parasite sampling, and histological examinations. (i) Experiment 1. Immune-related gene expression during primary AE.

To get a more complete picture on the influence of the parasite-induced host immune response following primary infection, the expression of 18 key cytokines and chemokines (5) was measured in the spleen at 21, 45, 60, and 120 days following egg infection, using qRT-PCR. Three independent experiments were carried out; each experimental group at each sampling time point included 6 animals. For one independent experiment, 48 female C57/BL6 mice aged between 8 and 10 weeks were randomly divided into two groups: the AE group (perorally infected with E. multilocularis eggs; see below) and the noninfected controls. At days 21, 45, 60, and 120 p.i., mice were euthanized in a CO_2_ chamber and spleens were harvested for total RNA extraction and subsequent qRT-PCR analyses. The mice with subcutaneous lesion and/or no lesion in the liver were excluded.

### (ii) Experiment 2. Foxp3^+^ Tregs as therapy target against primary AE.

To explore the potential role of Foxp3^+^ Tregs in protection against primary AE, Th1/Th2-related and Treg/Th17-related cytokines, antigen-presenting cell activation, and the generation of Tregs and their functions were studied at the different disease stages in an experimental model with active or depleted Foxp3^+^ Tregs. DEREG (depletion of regulatory T cells) mice of the C57/BL6 background with or without DT application, all aged between 8 and 10 weeks, were used to assess whether Foxp3 could be used as a target for treatment against AE. Age- and gender-matched littermates were used for mock-infected control groups. Each group included 6 animals if not stated otherwise, and the experiment was performed with 24 mice in total (divided into 4 groups, including AE-DEREG DT^+^, control-DEREG DT^+^, AE-DEREG DT^−^, and control-DEREG DT^−^). In DEREG mice, a diphtheria toxin (DT) receptor together with an enhanced green fluorescent protein (eGFP) is expressed under the control of the Foxp3 promoter. Therefore, depletion of Treg cells can be achieved by appropriate DT application (36). As shown elsewhere (37), in naive DEREG mice a subset of Foxp3^+^ Treg cells lacking the DT receptor (DTR) and GFP expression are not affected by the treatment. All animals were bred in-house, confirmed by genotyping for GFP and DTR genes (38), and additionally monitored by daily inspection, including the assessment of the appearance of health status and putative weight loss or gain during the whole course of the experiment.

### Parasite and experimental infection.

E. multilocularis eggs were isolated from a naturally E. multilocularis-infected dog, which was euthanized at the Small Animal Clinic of the Vetsuisse Faculty due to a noninfectious disease. Infection with E. multilocularis was detected upon routine necropsy investigation by pathologists. To prepare the parasite eggs for subsequent infection of mice, the dog intestine was removed under appropriate safety precautions and cut into 4 pieces. After longitudinal opening of the intestinal segments, the worm-containing mucus was scraped out and put into petri dishes containing sterile water. Subsequently, the mucosal suspension was serially filtered through a 500-μm and then a 250-μm metal sieve, by concurrently disrupting the worms with an inversed 2-ml syringe top. This suspension was further filtered through a 105-μm nylon sieve. The eggs were then washed by repeated sedimentation (1 × *g*, 30 min, room temperature) in sterile water containing 1% penicillin-streptomycin and stored in the same solution at 4°C. For primary infection of mice, animals received approximately 400 eggs suspended in 100 μl sterile water by peroral gavage. Control mice (mock infection) received 100 μl water only. All animal infections were performed in a biosafety level 3 unit (permit no. A990006/3).

### Treg cell depletion.

To explore whether Foxp3 could be putatively tackled as an immunotherapeutic target against primary E. multilocularis infection, E. multilocularis-infected DEREG mice with or without DT application and corresponding control WT littermates were investigated for the growth kinetics of the individually developing intrahepatic parasite lesions. DEREG-C57/BL6 mice (DEREG) and their WT littermate controls were used either as infected animals or as noninfected controls. All mice belonging to the Treg cell depletion group received 110 ng of DT i.p. (Calbiochem, Merck, Germany) dissolved in 100 μl phosphate-buffered saline (PBS) starting at 3 weeks postinfection and maintained for 4 weeks with a frequency of 3 applications/week. Successful depletion and rebound of Treg cells were confirmed by flow-cytometric analysis based on the detection of the FoxP3eGFP signal in splenic leukocytes. Infected mice were daily monitored for survival/well-being and morbidity.

### Parasite lesion quantification and spleen sampling.

All mice orally infected with E. multilocularis eggs and their noninfected controls were euthanized in a CO_2_ chamber at the end of DT application. At necropsy, the number and size (diameter) of the individual liver lesions (each caused by one developing oncosphere, originating from one parasite egg) were recorded. The mice with subcutaneous lesion and/or no lesion in the liver were excluded. Spleens were harvested according to the same protocol as that used in experiment 1, for both single spleen cell suspension and subsequent fluorescence-activated cell sorting (FACS) cell staining.

### Total RNA extraction and qRT-PCR.

Total RNA was isolated from spleen using the Qiagen RNeasy minikit according to the manufacturer's instructions. The quality of the isolated RNA was determined with a NanoDrop ND 1000 (NanoDrop Technologies) and a Bioanalyzer 2100 (Agilent). Only samples with a 260-nm/280-nm ratio between 1.9 and 2.1 and a 28S/18S ratio between 1.5 and 2 were further processed. cDNA was synthesized using the Omniscript reverse transcription kit (Qiagen, Hilden, Germany). SYBR green mix-based qRT-PCR was carried out on a Rotor-Gene 6000 qPCR detection system (Corbett) with the FastStart Essential DNA Green Master (Roche, Basel, Switzerland) as per the manufacturer's instructions. PCR cycling was performed in triplicates in final volumes of 20 μl containing 2 μl cDNA and 10 pM of each primer (cycle scheme: initial denaturation at 95°C for 15 min and 45 cycles of 95°C for 15 s, 55°C for 30 s, and 72°C for 30 s). Fluorescence was measured in every cycle, and a melting curve was analyzed after the PCR by increasing the temperature from 55 to 95°C in 0.5°C increments. The primers used were described earlier (5), and mRNA levels of different cytokines were quantified relative to the mRNA level of housekeeping gene *β-actin*.

### Cell preparations and flow cytometry.

Spleen cells from naive (control) and E. multilocularis-infected (AE) DEREG and WT mice were collected by grinding of spleens with 5 ml RPMI 1640 (Gibco). Cells were subsequently washed twice and resuspended in RPMI 1640 for cell staining or cell culture. Macrophages were removed from each group of mice by plastic adhesion as follows. Spleen cell suspensions were incubated in 15 ml RPMI 1640–20% fetal calf serum (FCS) in a petri dish for 2 h at 37°C, in humid atmosphere containing 5% CO_2_. Subsequent to incubation, nonadherent cells were separated from macrophage-enriched adherent cells, and this new cell suspension was used for FACS analyses.

Aliquots of 10^6^ cells/100 μl of staining buffer per well were each incubated with 1 μg of purified anti-CD16/CD32 for 20 min in the dark in order to block nonspecific binding of antibodies to the FcγIII and FcγII receptors. Subsequently, these cells were separately stained with the following surface markers for 15 min with 1 μg of primary antibodies: APC-labeled anti-CD4, anti-CD80, anti-CD86; phycoerythrin (PE)-labeled anti-CD11b and anti-CD11c. All antibodies were from eBioscience (San Diego, CA, USA). For intracellular staining, spleen cells were first incubated with Inside Fix (Miltenyi Biotec, Bergisch Gladbach, Germany) for 20 min at room temperature and subsequently stained with PE-labeled anti-IFN-γ, anti-IL-4, anti-IL-17A, and anti-IL-10 (eBioscience, San Diego, CA, USA) in Inside Perm (Miltenyi Biotec, Bergisch Gladbach, Germany) for 15 min in the dark. Corresponding fluorochrome-labeled isotype control antibodies were used for staining controls. Cells resuspended in 300 μl of buffer (0.15 M NaCl, 1 mM NaH_2_PO_4_·H_2_O, 10 mM Na_2_HPO_4_·2H_2_O, and 3 mM NaN_3_) were analyzed in a flow cytometer (Becton Dickinson, Heidelberg, Germany) using the corresponding Cell Quest software.

### Statistical analyses.

All data were analyzed by SPSS 17.0. The results are presented as means ± standard deviations (SD). Normality of data was assessed by the D'Agostino & Pearson and Shapiro-Wilk tests. For normally distributed groups of data, one-way analysis of variance (ANOVA) followed by Bonferroni's posttest was used to compare the differences between groups for cytokine gene expression (experiment 1), and unpaired two-tail Student's *t* test was used to compare the differences between groups for the lesion number and size (experiment 2). Significance was defined at *P* values of <0.05 for all tests except those with Bonferroni's correction.

## Supplementary Material

Supplemental file 1
