# Supplementary material for: Foxp3+ T Regulatory Cells as a Potential Target for Immunotherapy against Primary Infection with Echinococcus multilocularis Eggs
Source: Infect Immun. 2018 Sep 21;86(10):e00542-18. doi: 10.1128/IAI.00542-18 (PMC6204723; doi:10.1128/IAI.00542-18)
Supplement: Supplemental file 1 [file zii999092552s1.pdf]

Supplementary Figure 1. **Foxp3$^+$ Tregs inducible knock-down as a potential immunotherapeutical target against primary AE and Th2/Th17 related immune response (assessed by FACS)**

(A) Frequency of IL-4$^+$ T cells within CD4$^+$ T cells in spleen cells from AE-DEREG DT- and AE-DEREG DT+ mice at 3 months post-infection. (B) Representative images of IL-4$^+$ T cells within CD4$^+$ T cells in spleen cells from AE-DEREG DT- and AE-DEREG DT+ mice 3 months post-infection. (C) Frequency of IL-17A$^+$ T cells within CD4$^+$ T cells in spleen cells from AE-DEREG DT- and AE-DEREG DT+ mice. (D) Representative images of IL-17A$^+$ T cells within CD4$^+$ T cells in spleen cells from AE-DEREG DT- and AE-DEREG DT+ mice 3 months post-infection.

Data represent mean±SD of a total of 6 mice in non-infected control group, 5 mice in AE group (AE-DEREG DT- and AE-DEREG DT+) after excluding the mice with subcutaneous lesions or no lesion in the liver. Comparison between groups was performed using a two-way ANOVA with Bonferroni's multiple comparison post-test for statistical analysis. *$P<0.025$.

'DEREG DT-', *foxp3* inducible knock-down mice (DEREG mice) without DT application; 'DEREG DT+', DEREG mice with DT application; 'AE- DEREG DT-', *E. multilocularis*-infected DEREG without DT application; 'AE- DEREG DT+', *E. multilocularis*-infected DEREG mice with DT application. 'Control', non-infected mice. DT application started 1 month post infection, maintained for one month, the mice were sacrificed at 3 months post infection.

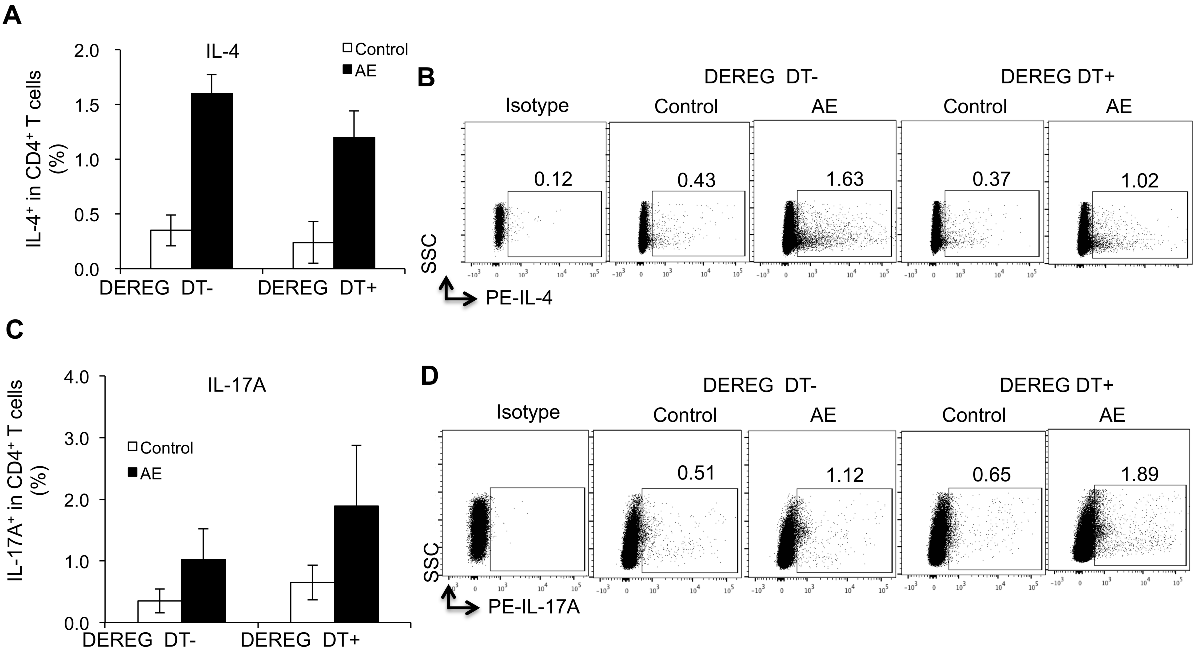
A
IL-4
Control
AE
IL-4+ in CD4+ T cells (%)
2.0
1.5
1.0
0.5
0.0
DEREG DT-
DEREG DT+
B
DEREG DT-
DEREG DT+
Isotype
Control
AE
Control
AE
0.12
0.43
1.63
0.37
1.02
SSC
PE-IL-4
C
IL-17A
Control
AE
IL-17A+ in CD4+ T cells (%)
4.0
3.0
2.0
1.0
0.0
DEREG DT-
DEREG DT+
D
DEREG DT-
DEREG DT+
Isotype
Control
AE
Control
AE
0.51
1.12
0.65
1.89
SSC
PE-IL-17A
